# Irisin Signaling Resistance in Myalgic Encephalomyelitis: A Proposed Mechanistic Framework for Post-Exertional Malaise Involving the TSP-1–HSP90α–αvβ5 Axis

**DOI:** 10.3390/ijms27114770

**Published:** 2026-05-26

**Authors:** Bernard Souma, Wesam Elremaly, Marie-Yvonne Akoume, Mohamed Elbakry, Christian Godbout, Alain Moreau

**Affiliations:** 1 Viscogliosi Laboratory in Molecular Genetics of Musculoskeletal Diseases, Azrieli Research Center, CHU Sainte-Justine, Montreal, QC H3T 1C5, Canada; 2 Department of Biochemistry and Molecular Medicine, Faculty of Medicine, Université de Montréal, Montreal, QC H3T 1J4, Canada; 3 Open Medicine Foundation ME/CFS Collaborative Center, CHU Sainte-Justine/Université de Montréal, Montreal, QC H3T 1C5, Canada; 4 Patient-Partner, ICanCME Research Network, Azrieli Research Center, CHU Sainte-Justine, Montreal, QC H3T 1C5, Canada; 5 Department of Cellular and Molecular Biology and Genetics, Institut Supérieur de Biologie Médicale (ISBM), Université des Sciences de la Santé (USS) de Libreville, Libreville P.O. Box 18231, Gabon; 6 Biochemistry Section, Chemistry Department, Faculty of Science, Tanta University, Tanta 31527, Egypt; 7 Department of Stomatology, Faculty of Dentistry, Université de Montréal, Montreal, QC H3T 1J4, Canada

**Keywords:** myalgic encephalomyelitis (ME), post-exertional malaise (PEM), irisin, thrombospondin-1 (TSP-1), αvβ5 integrin, HSP90α, energy metabolism, cell signaling

## Abstract

Myalgic Encephalomyelitis (ME) is a chronic, multisystem disease characterized by systemic metabolic dysfunction and post-exertional malaise (PEM). In this study, we investigated the dysregulation of irisin, an exercise-induced myokine, and its potential antagonism by thrombospondin-1 (TSP-1). In a cross-sectional study (92 ME patients vs. 44 sedentary healthy controls), plasma irisin and TSP-1 levels were measured at baseline and after a 90 min mechanical stress challenge applied to induce PEM. ME patients exhibited significantly lower baseline irisin (*p* < 0.05) and a blunted exertional response (*p* < 0.05). Paradoxically, baseline irisin was an independent predictor of fatigue severity (β = 0.728, *p* = 0.018), with moderate-to-severe patients showing elevated levels of both irisin and TSP-1 (*p* < 0.05), suggesting a compensatory but ineffective response. Functional cellular dielectric spectroscopy indicated that TSP-1 inhibits irisin signaling in a concentration-dependent manner. Irisin signaling was markedly reduced by both αvβ5 blockade and HSP90α inhibition in this experimental system, consistent with a diminished ability to counteract TSP-1. Collectively, these findings support a model in which dysregulation of the irisin–TSP-1 axis contributes to metabolic dysfunction in ME. Elevated circulating TSP-1 levels are associated with symptom severity and are linked to impaired irisin signaling in an HSP90α- and αvβ5-dependent context. This interaction is consistent with defective metabolic adaptation and highlights a potential therapeutic target that warrants further validation to restore energy homeostasis.

## 1. Introduction

Myalgic encephalomyelitis (ME) is a complex, multisystem, and disabling disorder characterized by persistent, unexplained fatigue, post-exertional malaise (PEM), and a constellation of cognitive, autonomic, and neurological impairments [1,2,3]. PEM, the clinical hallmark of ME, is defined as a delayed and disproportionate exacerbation of symptoms following minimal physical or mental exertion, often persisting for days to weeks [4]. Despite its substantial socio-economic burden and profound impact on quality of life, the underlying pathophysiological mechanisms of ME remain poorly defined, and the disease lacks both validated molecular diagnostic tools and effective disease-modifying therapies [5].

Accumulating evidence implicates disrupted energy metabolism and mitochondrial dysfunction as central features of ME pathogenesis. Patients consistently demonstrate reduced oxidative phosphorylation capacity and impaired cellular bioenergetic responses, which may underlie their diminished resilience to metabolic stress [6,7,8]. Within this context, irisin, a 112-amino acid myokine derived from the proteolytic cleavage of fibronectin type III domain-containing protein 5 (FNDC5), has emerged as a key regulator of metabolic homeostasis. Induced by peroxisome proliferator-activated receptor gamma coactivator 1-alpha (PGC1-α) during exercise, irisin promotes the browning of white adipose tissue, enhances mitochondrial thermogenesis, and facilitates glucose uptake [9,10]. Given that irisin expression is closely linked to physical activity and exercise-induced metabolic adaptation, reduced physical activity levels commonly observed in ME patients may also contribute, at least in part, to lower circulating irisin levels. In addition, irisin has demonstrated neuroprotective and anti-fatigue effects in several neurological conditions [11,12,13]; however, its role and signaling integrity in ME remain unexplored.

While irisin represents a critical adaptive metabolic signal, parallel pathways associated with vascular and inflammatory dysfunction may counteract these beneficial effects. Specifically, the matricellular protein thrombospondin-1 (TSP-1) is reported to be elevated in ME patient cohorts [14], reflecting underlying platelet hyperactivation and endothelial dysfunction. As a multifunctional glycoprotein, TSP-1 inhibits nitric oxide (NO) signaling and angiogenesis, potentially exacerbating tissue hypoxia and metabolic dysregulation [15]. Crucially, TSP-1 engages multiple cell-surface receptors, including integrin-associated pathways [16], which are also implicated in mediating irisin signaling, particularly within the αV integrin family [17,18]. In addition, extracellular heat shock protein 90 alpha (HSP90α) has emerged as an important regulator of integrin activation and receptor-mediated signaling. Unlike the predominantly intracellular HSP90β isoform, HSP90α can be secreted into the extracellular environment, where it modulates ligand–receptor interactions and stress-response signaling pathways. Previous studies have demonstrated that extracellular HSP90α participates in the regulation of αv integrin-dependent signaling complexes, providing a mechanistic rationale to investigate its potential involvement in irisin-mediated metabolic signaling [19,20,21]. This shared receptor interface raises the possibility of competitive or reciprocal modulation between these pathways [16,17,18]. However, despite the recognized roles of irisin in metabolic adaptation and TSP-1 in chronic disease pathology, their functional interaction remains poorly understood and has not been investigated in the context of ME. In particular, it remains unclear whether elevated TSP-1 may antagonize irisin signaling by interfering with receptor engagement and downstream metabolic responses.

This knowledge gap is particularly relevant to the pathophysiological paradox observed in ME, in which physical exertion fails to elicit adaptive metabolic responses and instead precipitates symptom exacerbation [22]. The present study aims to define the functional role of irisin in ME pathogenesis and elucidate the molecular mechanisms underlying its impaired signaling. We hypothesized that elevated TSP-1 acts as a molecular inhibitor of irisin signaling by interfering with αvβ5 integrin binding and disrupting its critical interaction with the chaperone protein HSP90α. To test this hypothesis, we employed a cross-sectional clinical design combined with cellular dielectric spectroscopy (CDS) to characterize, in real time, the dynamic interactions between irisin and TSP-1. By elucidating the irisin–TSP-1 signaling axis, this study aims to provide a comprehensive mechanistic framework for PEM and to identify specific therapeutic targets for restoring metabolic homeostasis in ME.

## 2. Results

### 2.1. Clinical and Demographic Profiles of the Study Cohort

The study population comprised 92 patients diagnosed with ME according to the Canadian Consensus Criteria (CCC) and 44 sedentary healthy controls (HCs). No significant differences in age were observed between the groups, with a mean of 49 ± 1.2 years in ME patients and 50 ± 1.6 years in HCs. Consistent with the known epidemiology of the disease, females predominated in both cohorts, representing 77.2% of the ME group and 56.8% of the HC group. Body mass index (BMI) was comparable between groups (26 ± 0.6 kg/m^2^ in ME vs. 25 ± 0.7 kg/m^2^ in HCs). Age- and BMI-matching between groups was implemented to reduce potential confounding effects related to demographic and anthropometric variables. Clinical assessments confirmed a substantial disease burden in the ME cohort. Short Form-36 (SF-36) scores for physical health were significantly lower in ME patients compared to HCs, with similar impairments observed in mental health components. Furthermore, Multidimensional Fatigue Inventory (MFI-20) and DePaul Symptom Questionnaire (DSQ) scores indicated that ME patients exhibited significantly higher levels of general fatigue, cognitive dysfunction, and PEM than controls (*p* < 0.0001; Table 1).

### 2.2. Dysregulation of Circulating Irisin and Response to Exertion

**Figure 1:**
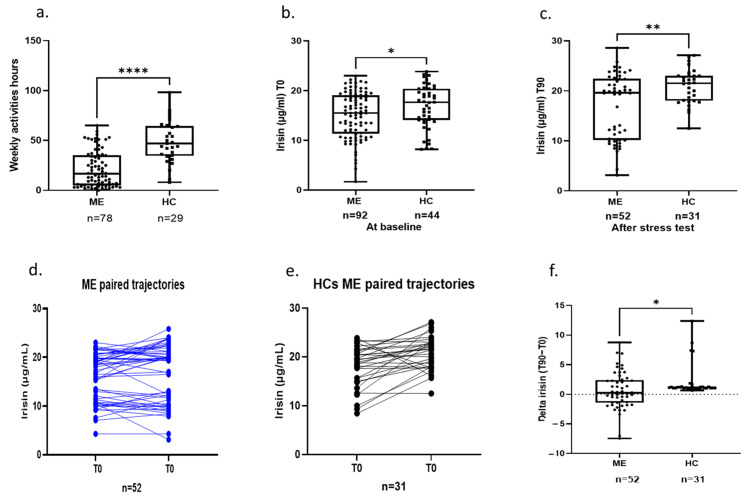
Comparative analysis of circulating irisin levels and exertional response in ME patients and healthy controls. (**a**) Self-reported weekly hours of physical activity were markedly lower in the ME (*n* = 78) cohort than in sedentary healthy controls (HCs; *n* = 29). (**b**) Baseline (T0) circulating irisin levels were significantly lower in patients with ME (*n* = 92) compared to HCs (*n* = 44). (**c**) Circulating irisin levels following a standardized 90 min (T90) mechanical stress challenge, performed in a subset of participants (ME: *n* = 52; HC: *n* = 31). Although irisin levels increased from baseline in both groups, the response was significantly attenuated in ME patients. (**d**,**e**) Paired individual trajectories of circulating irisin levels before (T0) and following a standardized 90 min mechanical stress challenge (T90), performed in a subset of participants (ME: *n* = 52; HC: *n* = 31). (**f**) The change in circulating irisin levels (∆T90–T0), representing the absolute difference between post-stress and baseline values, was significantly smaller in the ME group compared to HCs (mean difference = 1.477 ± 0.6847, 95% CI: 0.1149 to 2.839, *p* = 0.0339). Data are presented as box plots showing the median and individual data points. Statistical significance for independent groups was assessed using independent *t*-tests. Data normality was verified using the Shapiro–Wilk test. Significance levels are indicated as follows: * *p* < 0.05; ** p < 0.01; **** p < 0.0001.

Analysis of the study cohort and plasma samples revealed significant alterations in circulating irisin dynamics. Consistent with the clinical burden of the disease, self-reported weekly physical activity hours were markedly reduced in the ME cohort relative to HCs (*p* < 0.0001; Figure 1a). At baseline (T0), circulating irisin levels were significantly lower in patients with ME (*n* = 92) compared to HCs (*n* = 44, *p* = 0.03; Figure 1b). To assess the immediate response to exertion, a subset of participants (ME: *n* = 52; HC: *n* = 31) underwent a standardized 90 min mechanical stress challenge (Figure 1c). Paired individual trajectory analyses further illustrated the heterogeneous within-subject irisin responses following exertion in both ME patients and HCs (Figure 1d,e). Circulating irisin levels increased from baseline in both groups; however, the magnitude of this response, quantified as the change in circulating irisin concentration (∆ irisin; T90–T0), was significantly reduced in ME patients compared to HCs (mean difference = 1.477 ± 0.684, 95% CI: 0.114 to 2.839, *p*= 0.0339; Figure 1f). Collectively, these findings indicate an impaired capacity to appropriately regulate irisin in response to physiological stress in ME.

### 2.3. Sex-Specific Differences in Circulating Irisin and Correlations with PEM Severity

**Figure 2:**
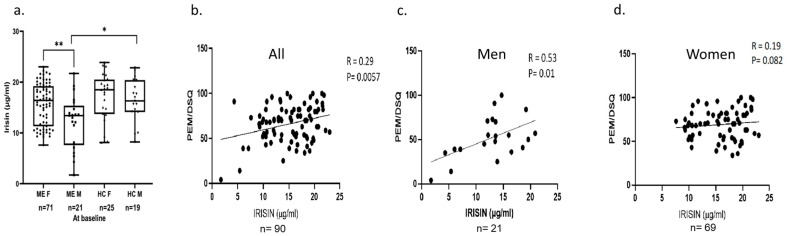
Sex-specific comparisons of circulating irisin levels and correlations with PEM severity (**a**) Baseline comparison of circulating irisin levels between female (F) and male (M) participants. A significant deficit was observed in male ME patients compared to female ME patients (** *p* < 0.01), whereas no significant difference was found between sexes in the healthy controls (HCs) group. Data are presented as box plots showing the median and individual data points. (**b**) Scatter plot showing a significant positive correlation between baseline circulating irisin levels and DSQ-PEM scores in the total ME cohort (*n* = 90, R = 0.29, *p* = 0.0057, FDR-adjusted *p* = 0.01). (**c**) Correlation analysis in male ME patients (*n* = 21) demonstrates a significant association between irisin levels and PEM severity (R = 0.53, *p* = 0.01, FDR-adjusted *p* = 0.02). (**d**) Correlation analysis in female ME patients (*n* = 69) showed no statistically significant association between the variables (R = 0.19, *p* = 0.082, FDR-adjusted *p* = 0.16). Group comparisons in Panel (**a**) were performed using unpaired two-tailed Student’s *t*-tests. Correlations in Panels (**b**–**d**) were assessed using Pearson’s correlation coefficients. Of note, p-values were adjusted for multiple comparisons using the Benjamini–Hochberg false discovery rate (FDR) method. Significance levels are indicated as follows: * *p* < 0.05; ** *p* < 0.01.

Comparative analysis at T0 revealed significant sex-based differences in circulating irisin levels within the ME cohort. Female patients (*n* = 71) exhibited significantly higher irisin levels than male patients (*n* = 21; *p* < 0.01; Figure 2a). In contrast, no significant differences were observed between female HCs (*n* = 25) and male HCs (*n* = 19), indicating that this sex-dependent divergence is specific to the disease state. Despite these baseline differences, a positive correlation was identified between circulating irisin levels and PEM severity, as assessed by the DSQ, across the entire ME cohort (R = 0.29, *p* = 0.0057, FDR-adjusted *p* = 0.01; Figure 2b). Stratified analyses further indicated that this association differed markedly by sex. In male patients, irisin levels were significantly correlated with PEM severity (R = 0.53, *p* = 0.01, FDR-adjusted *p* = 0.02; Figure 2c), whereas no significant association was observed in female patients (R = 0.19, *p* = 0.082, FDR-adjusted *p* = 0.16; Figure 2d). These findings suggest that the relationship between irisin signaling and symptom exacerbation is modulated by sex-specific biological mechanisms, highlighting a potential dimorphism in the molecular drivers of PEM.

### 2.4. Fatigue Severity-Dependent Dysregulation of the Irisin–TSP-1 Axis

**Figure 3:**
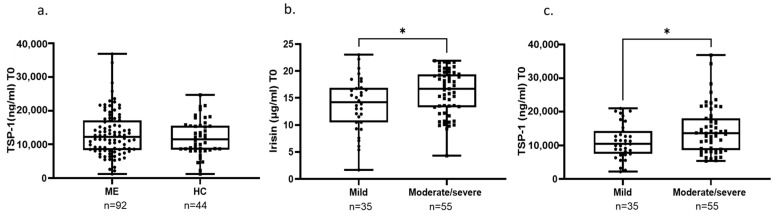
Stratification of circulating irisin levels and TSP-1 levels by fatigue severity in ME patients. (**a**) Baseline circulating TSP-1 levels (ng/mL) compared between the total ME cohort (*n* = 92) and healthy controls (HC, *n* = 44). No significant differences were observed between diagnostic groups at baseline. (**b**) Comparative analysis of baseline irisin levels (µg/mL) stratified by Multidimensional Fatigue Inventory (MFI-20) total scores. (**c**) Comparative analysis of baseline circulating TSP-1 levels (ng/mL) across fatigue severity groups. Patients with moderate-to-severe fatigue (scores 76–100, *n* = 55) exhibited significantly higher circulating levels of both markers compared to those with mild fatigue (scores 51–75, *n* = 35) (*p* < 0.05). Data are presented as box plots showing the median and individual data points. Comparisons were performed using independent *t*-tests. Data normality was verified using the Shapiro–Wilk test. Statistical significance is indicated by * *p* < 0.05.

To investigate whether components of the irisin–TSP-1 axis reflect disease severity, the ME cohort was stratified based on total MFI-20 scores into mild fatigue (51–75; *n* = 35) and moderate-to-severe fatigue (76–100; *n* = 55) groups. Unlike circulating irisin, which was significantly lower in the overall ME cohort at T0, circulating TSP-1 levels did not differ between ME patients and HCs at T0 (*p* = 0.35; Figure 3a) or T90. However, stratification by fatigue severity within the ME cohort revealed a distinct pattern: patients with moderate-to-severe fatigue exhibited significantly higher baseline levels of irisin (mean difference = 2.29 µg/mL, 95% CI: 0.31 to 4.28, Cohen’s d = 0.51, *p* = 0.024; Figure 3b) and TSP-1 (mean difference = 3351 ng/mL, 95% CI: 625 to 6078, Cohen’s d = 0.53, *p* = 0.017; Figure 3c) compared to those with mild fatigue. This parallel increase in both the myokine and its potential inhibitor among the most severely affected patients is suggestive of a compensatory but potentially insufficient metabolic response to increased physiological stress. Importantly, these findings indicate that TSP-1 is associated with disease severity within ME rather than serving as a distinguishing marker between ME patients and HCs.

To determine whether medication exposure or comorbidity burden contributed to the observed TSP-1 alterations, additional subgroup analyses were performed across the major medication classes and comorbidity categories identified within the cohort. Medications previously reported to influence inflammatory, vascular, metabolic, or fibrotic pathways associated with TSP-1 biology including corticosteroids, hormone-related therapies, aspirin, metformin, gabapentinoids, angiotensin-related medications, and vitamin D supplementation did not significantly alter circulating TSP-1 levels at T0, T90, or in the delta response following the provocation maneuver. Similarly, no significant differences in circulating TSP-1 levels were observed according to comorbidity status, including autoimmune/inflammatory disorders (including fibromyalgia), cardiovascular/autonomic disorders, endocrine/metabolic disorders, respiratory/allergic disorders, gastrointestinal/hepatic disorders, or neurological/neuropsychiatric conditions. Collectively, these findings indicate that the observed TSP-1 alterations were not driven by medication exposure, polypharmacy, or overlapping comorbid conditions within the cohort.

### 2.5. Independent Predictors of Fatigue Severity

To confirm that these severity-dependent elevations were not confounded by the previously noted sex-specific differences or other demographic variables, we evaluated these relationships using a multivariable regression approach. The total MFI-20 score was used as the dependent variable, with baseline irisin, age, sex, BMI, and disease duration as independent predictors; sex was coded as a binary variable (female = 1, male = 2). The analysis demonstrated that baseline irisin was the only significant independent predictor of fatigue severity (*p* = 0.021), with higher plasma irisin levels associated with increased fatigue scores (β = 0.670, 95% CI: 0.106 to 1.235; Table 2). In contrast, age (*p* = 0.829), sex (*p* = 0.465), BMI (*p* = 0.350), and disease duration (*p* = 0.886) were not significantly associated with fatigue severity. The regression model explained approximately 10.5% of the variance in fatigue severity (R^2^ = 0.1048; *n* = 87). Diagnostic evaluation confirmed the robustness of the model, with normally distributed residuals (Shapiro–Wilk *p* = 0.0631), constant variance of residuals (homoscedasticity) as verified by visual inspection of residual versus predicted plots, and no evidence of multicollinearity among predictors (all VIF < 1.4). Collectively, these findings indicate that elevated circulating irisin levels are independently associated with increased fatigue severity in ME, supporting a model in which increased irisin may reflect a compensatory but functionally ineffective metabolic response, although the overall variance explained by the model remains modest, potentially driven by a concurrent rise in the antagonist TSP-1.

A multiple linear regression model was constructed to evaluate the independent association between circulating irisin and fatigue severity in patients with ME (*n* = 87). The total score of the MFI-20 questionnaire was used as the dependent variable. Baseline irisin levels (T0), sex, BMI, age, and disease duration were included as independent predictors. Statistical significance of individual regression coefficients was assessed using *t*-tests. Multicollinearity was evaluated using variance inflation factors (VIFs), with values < 1.4 indicating no significant collinearity among predictors. Model residuals were assessed for normality using the Shapiro–Wilk test (*p* = 0.063).

### 2.6. Functional Molecular Interactions and Receptor Dynamics of the Irisin–TSP-1 Axis

**Figure 4:**
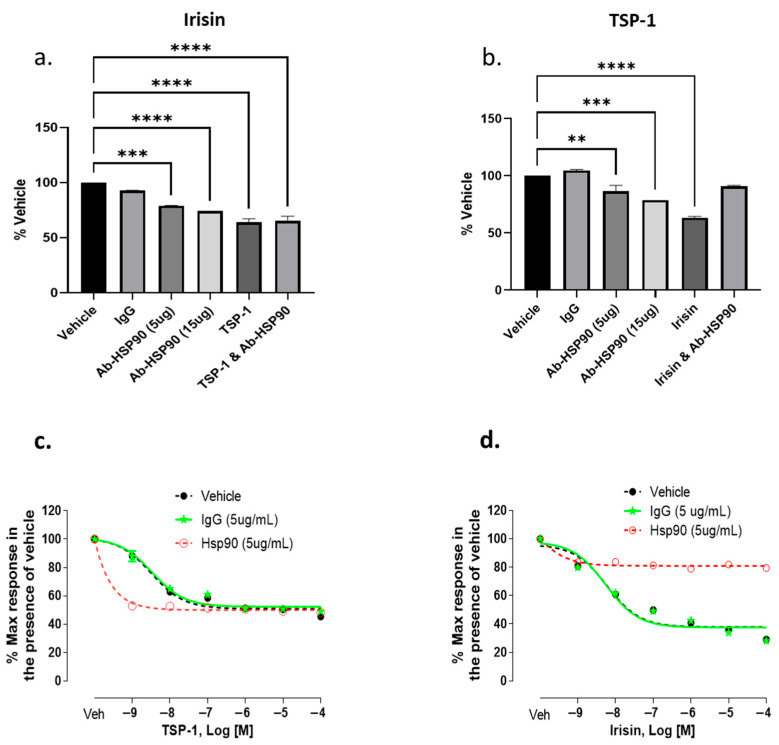
Functional signaling interactions between molecular irisin and TSP-1 mediated by HSP90α. (**a**) Irisin-induced cellular responses measured by Cellular Dielectric Spectroscopy (CDS). Pre-treatment with HSP90α-specific antibodies or recombinant TSP-1 significantly reduced irisin bioactivity. (**b**) TSP-1-induced responses showed relative independence from HSP90α inhibition compared to irisin. (**c**) Concentration-dependent inhibition of irisin signaling by TSP-1. (**d**) Concentration-dependent inhibition of TSP-1 signaling by irisin. Concentration-response curves were analyzed using nonlinear regression (sigmoidal dose–response). Differences between treatment conditions in Panels (**a**) and (**b**) were assessed using one-way ANOVA followed by Tukey’s multiple comparisons test. Statistical significance bars are shown for the principal comparisons relative to the vehicle condition. Data are presented as mean ± SD from three independent biological replicates (*n* = 3) and are expressed as a percentage of the maximum response relative to the vehicle control. Significance levels are indicated as follows: ** *p* < 0.01; *** *p* < 0.001; **** *p* < 0.0001.

To elucidate the molecular mechanisms underlying the observed alterations in circulating biomarkers and signaling responses in ME, we investigated the crosstalk between irisin and TSP-1 using CDS, a label-free, real-time assay of receptor-mediated cellular responses based on impedance measurements. We focused on HSP90α based on evidence that, unlike the predominantly intracellular HSP90β isoform, HSP90α can be secreted extracellularly, where it modulates ligand–receptor interactions and integrin activation [20,21]. Notably, extracellular HSP90α has been shown to regulate integrin activation, including αvβ5, and more recent studies indicate that irisin signals through αv integrins, providing a mechanistic rationale to examine its role in this system [18]. Accordingly, we used Jurkat T cells as a well-established model for integrin-dependent signaling and impedance-based assays. Stimulation of Jurkat cells with recombinant irisin induced a robust impedance-based cellular response, which was significantly attenuated by an HSP90α-specific neutralizing antibody in a dose-dependent manner (Figure 4a). This reduction, observed at both 5 µg and 15 µg concentrations, indicates that irisin bioactivity in this CDS assay is at least partly dependent on extracellular HSP90α-mediated signaling.

Furthermore, pre-treatment with TSP-1 (10^−6^ M) inhibited irisin-induced responses by approximately 40% (Figure 4a). The most pronounced suppression occurred under simultaneous HSP90α blockade, where the inhibitory effect of TSP-1 on irisin was further amplified (Figure 4a,c). Conversely, although recombinant TSP-1 protein induced a measurable cellular response, its activity remained largely independent of HSP90α inhibition (Figure 4b), suggesting the involvement of alternative signaling pathways that do not rely on this chaperone. Reciprocal dose–response analysis demonstrated that TSP-1 inhibits irisin signaling in a concentration-dependent manner (Figure 4c). Notably, while irisin was capable of inhibiting TSP-1 activity under vehicle conditions, this inhibitory capacity was markedly reduced following HSP90α neutralization (Figure 4d). These findings indicate a reciprocal antagonistic relationship in which the functional efficacy of irisin appears to be dependent on the availability of HSP90α in this experimental system.

**Figure 5:**
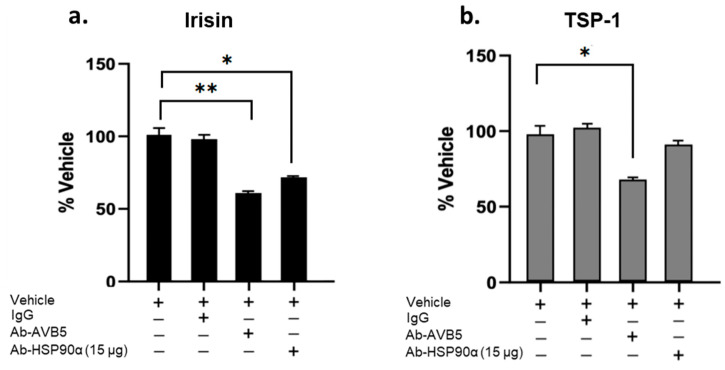
Differential dependency of irisin and TSP-1 signaling on αVβ5 integrin and HSP90α. (**a**) Irisin-induced cellular responses measured by Cellular Dielectric Spectroscopy (CDS). Bioactivity was significantly attenuated by both the αvβ5-specific antibody (Ab-AVB5) and the HSP90α-specific antibody (Ab-HSP90α, 15 µg), demonstrating a dual dependency on the receptor and its cofactor. (**b**) TSP-1-induced responses showed partial reduction with αvβ5 blockade and minimal sensitivity to HSP90α inhibition, suggesting the involvement of alternative signaling pathways. Differences between conditions were assessed using one-way ANOVA followed by Tukey’s multiple comparisons test. Statistical significance bars are shown for the principal comparisons relative to the vehicle condition. Data are presented as mean ± SD from three independent biological replicates (*n* = 3) and are expressed as a percentage of the maximum response relative to the vehicle and IgG controls. Significance levels are indicated as follows: * *p* < 0.05; ** *p* < 0.01.

The involvement of the αvβ5 integrin was further examined using competitive inhibition assays (Figure 5). Pre-treatment with an αvβ5-specific antibody significantly reduced irisin-induced responses relative to vehicle levels (Figure 5a). This degree of inhibition was comparable to that observed with HSP90α blockade (15 µg), indicating that irisin signaling in this system is sensitive to both αvβ5 blockade and HSP90α inhibition. In contrast, TSP-1 signaling exhibited only partial dependency on αvβ5 (Figure 5b) and minimal sensitivity to HSP90α inhibition, indicating the use of alternative signaling pathways. Collectively, these findings support a model in which elevated TSP-1 may act as a functional inhibitor of irisin signaling within a signaling context sensitive to both αvβ5 blockade and HSP90α inhibition.

## 3. Discussion

This study provides a novel molecular framework for understanding the energy metabolism dysfunction and PEM that characterize ME. By combining clinical data with bioimpedance assays, we suggest dysregulation of the irisin–TSP-1 axis. Our findings suggest that the metabolic impairment observed in ME is not solely attributable to reduced irisin production, but rather reflects a complex biochemical antagonism in which elevated TSP-1 may act as a functional inhibitor of irisin-mediated signaling.

At the clinical level, ME patients exhibited significantly lower baseline circulating irisin levels compared with healthy controls. Given irisin’s established role in promoting mitochondrial oxidative capacity and glucose metabolism, this reduction is consistent with prior evidence of impaired pyruvate dehydrogenase activity and systemic bioenergetic dysfunction in ME [7]. More importantly, we observed a marked decoupling between irisin dynamics and physical exertion. Whereas healthy individuals demonstrate a robust increase in circulating irisin following physiological stress or exercise, ME patients display a blunted response that fails to meet the metabolic demands of physiological stress. This impaired responsiveness provides a potential molecular correlate of PEM, in which exertion fails to trigger adaptive metabolic recovery. Given the established exercise-dependent regulation of FNDC5/irisin expression, reduced physical activity levels commonly observed in ME patients may contribute, at least in part, to lower circulating irisin levels. However, the persistence of impaired irisin responsiveness following the standardized mechanical stress challenge, together with the observed dysregulation of the irisin–TSP-1 axis and the functional findings demonstratingTSP-1-mediated antagonism of irisin signaling within an HSP90α-sensitive and αvβ5-dependent context, supports the presence of broader intrinsic abnormalities affecting metabolic adaptation pathways in ME.

Furthermore, sex-specific differences in baseline irisin levels and their relationship with symptom severity point to biologically distinct mechanisms in ME. In male patients, higher baseline irisin levels were strongly associated with greater PEM severity, a relationship not observed in females. This sex-dependent pattern suggests that the contribution of irisin to symptom exacerbation differs by sex, potentially reflecting underlying differences in immune and metabolic regulation. However, given the smaller number of male participants, these sex-stratified correlations should be interpreted cautiously and require validation in larger independent cohorts. Accordingly, these findings should presently be considered exploratory and hypothesis-generating rather than definitive evidence of sex-specific biological mechanisms. While irisin has been extensively studied as an exercise-induced myokine involved in energy homeostasis, thermogenesis, and metabolic adaptation, its role in ME and, more broadly, in pathological responses to exertion remains largely unexplored. To our knowledge, no prior studies have examined the interplay between irisin and TSP-1 or assessed how this axis may influence receptor-mediated signaling pathways relevant to symptom exacerbation. In this context, our findings identify a previously unrecognized irisin–TSP-1 signaling axis and suggest that its dysregulation contributes to impaired metabolic adaptation in ME. Collectively, these results extend current understanding of irisin biology beyond physiological exercise responses and highlight a novel, potentially sex-dependent mechanism that may underlie heterogeneity in disease severity and progression [23,24,25].

Importantly, these findings support the concept that fatigue and PEM represent distinct, though overlapping, pathophysiological dimensions of ME. While PEM appears to be influenced by sex-specific biological factors, fatigue severity is independently associated with circulating irisin levels, indicating partially divergent underlying mechanisms. Stratification by disease severity revealed a paradoxical pattern: patients with moderate-to-severe fatigue exhibited higher baseline irisin levels than those with milder disease. This observation is consistent with a compensatory yet ineffective metabolic response, in which increased myokine production fails to translate into functional signaling. The robustness of this relationship is supported by multivariable regression analysis, which identified baseline irisin as an independent predictor of fatigue severity after adjustment for age, sex, BMI, and disease duration.

Mechanistically, this apparent disconnect between circulating irisin levels and functional signaling is explained by the antagonistic interaction between irisin and TSP-1 at the cell surface. CDS analyses demonstrate that TSP-1 inhibits irisin-induced signaling in a concentration-dependent manner within a context sensitive to αvβ5 blockade and HSP90α inhibition. In contrast, irisin signaling requires both αvβ5 engagement and the availability of HSP90α, consistent with the role of extracellular HSP90α in supporting receptor-mediated metabolic signaling [19]. Notably, TSP-1 retains its inhibitory activity even when these regulatory components are disrupted, highlighting its signaling dominance. In line with these mechanistic findings, increases in circulating TSP-1 were associated with disease burden rather than diagnostic status, although overall TSP-1 levels were comparable between ME patients and healthy controls, individuals with more severe fatigue exhibited significantly elevated levels of this antagonist. This imbalance is consistent with chronic platelet and endothelial activation reported in ME [26], where elevated matricellular proteins contribute to a pro-inflammatory milieu that suppresses adaptive metabolic responses [27]. Importantly, neutralization of HSP90α abolishes irisin’s ability to counteract TSP-1, effectively eliminating its protective effects and locking cells into a state of metabolic inactivity, as previously described in PEM models [7,28]. Together, these results support a model in which increasing disease severity is associated with a shift toward signaling resistance, in which elevated TSP-1 functionally overrides compensatory increases in irisin. In addition to TSP-1–mediated antagonism, emerging evidence suggests that alterations in the extracellular receptor landscape may further contribute to this state of signaling resistance. Recent work by Moezzi et al. reported elevated circulating levels of soluble low-density lipoprotein receptor-related protein 1 (LRP1/CD91) in a subset of ME patients [4], consistent with increased receptor shedding and extracellular remodeling. While membrane-bound LRP1 is a known receptor for extracellular HSP90α and participates in receptor-mediated signaling, its soluble form retains ligand-binding capacity and may act as a decoy by sequestering extracellular signaling partners. Although direct interactions between soluble LRP1 and irisin or HSP90α have not yet been demonstrated, increased circulating LRP1 could plausibly alter the availability or spatial organization of signaling complexes at the cell surface. In this context, elevated soluble LRP1 may further destabilize irisin-dependent signaling by perturbing HSP90α-integrin interactions or broader extracellular chaperone networks, thereby reinforcing the shift toward signaling resistance observed in ME.

**Figure 6:**
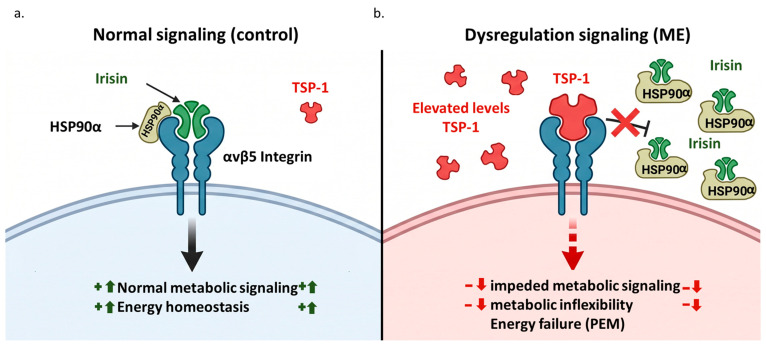
Proposed model of impaired irisin responsiveness in ME involving TSP-1, αvβ5 integrin, and extracellular HSP90α-dependent mechanisms. (**a**) Under physiological conditions, irisin engages αv integrins (including αvβ5) to trigger receptor-mediated signaling responses that are detectable by CDS. This signaling is dependent on both integrin engagement and the availability of extracellular HSP90α, which facilitates receptor activation and downstream signaling, thereby supporting adaptive metabolic responses to exertion and energy homeostasis. (**b**) In ME, elevated circulating TSP-1 contributes to a pathological extracellular environment that functionally suppresses irisin responsiveness. Through competitive and/or inhibitory interactions at the level of αv integrins and associated signaling complexes, TSP-1 shifts the balance toward reduced receptor activation, resulting in impaired metabolic adaptation associated with post-exertional malaise (PEM). The red “X” symbol indicates functional inhibition/blockade of irisin–HSP90α-mediated αvβ5 integrin signaling by elevated TSP-1. Red arrows indicate reduced or impaired signaling activity, whereas black arrows indicate normal downstream signaling. Green arrows and symbols represent preserved or enhanced metabolic homeostasis/signaling under physiological conditions, while red arrows and symbols represent impaired metabolic signaling and energy dysfunction in ME. Blue shading represents physiological/normal conditions, whereas red shading represents the pathological extracellular environment associated with ME. This inhibitory effect persists even under conditions where integrin or HSP90α-dependent signaling is disrupted, suggesting a dominant antagonistic role for TSP-1.

From a broader physiological perspective, the dysregulated irisin–TSP-1 axis may help explain the paradoxical response to exertion observed in ME. Under normal conditions, exercise induces irisin, which supports mitochondrial biogenesis, enhances oxidative phosphorylation, and promotes glucose uptake [29]. As illustrated in Figure 6, irisin signaling is proposed to involve mechanisms sensitive to both αvβ5 and HSP90α to support this energy homeostasis. However, our findings suggest that elevated TSP-1, particularly in more severe cases, may disrupt this adaptive response by interfering with irisin signaling at or upstream of the receptor level. This model is consistent with the clinical paradox observed in our cohort, where patients with moderate-to-severe fatigue exhibit higher baseline irisin levels that fail to alleviate symptoms, potentially due to concurrent TSP-1–mediated signaling interference. As a result, cells may exhibit a reduced capacity to adapt efficiently to metabolic stress, leading to decreased bioenergetic flexibility and delayed recovery following exertion. Given the established role of irisin in supporting mitochondrial respiration and metabolic flexibility [30], persistent TSP-1-mediated inhibition of irisin signaling could potentially contribute to impaired oxidative phosphorylation and altered metabolic adaptability under physiological stress conditions [31]. Future studies combining Seahorse metabolic flux analyses with modulation of the irisin–TSP-1 axis in PBMCs, skeletal muscle-derived cells, or patient-derived cellular systems will help determine whether this signaling dysregulation contributes to impaired mitochondrial respiration or altered extracellular acidification responses in ME. In addition, TSP-1 impairs NO signaling and vascular function, which may contribute to tissue hypoxia and abnormal perfusion [32]. These effects likely further aggravate metabolic dysfunction during and after exertion. Importantly, irisin signaling depends on both αvβ5 integrin engagement and HSP90α availability, revealing a key vulnerability in this pathway. Disruption of HSP90α- and αvβ5-sensitive signaling pathways not only weakens essential metabolic signaling but also allows TSP-1 activity to proceed unchecked. This shifts the cellular environment toward a pro-inflammatory and metabolically restrictive state. Together, these mechanisms provide a plausible basis for a state of impaired metabolic adaptability in ME [22,33], in which adaptive responses fail to activate despite increased physiological demand, sustaining a cycle of energy deficit and symptom worsening.

From a translational perspective, the irisin–TSP-1 axis represents a promising therapeutic target. Strategies that reduce TSP-1 levels, block its receptor interactions, or restore NO signaling could help relieve this inhibitory molecular brake. At the same time, approaches aimed at stabilizing or enhancing HSP90α-dependent irisin signaling such as small-molecule chaperones or protease inhibitors, may restore metabolic responsiveness to stress. Ultimately, these interventions could help re-establish the link between physical activity and adaptive energy metabolism, improving clinical outcomes and quality of life in ME. In parallel, our findings support the potential use of irisin–TSP-1 profiling as a stratification tool to identify metabolic endophenotypes in ME, thereby guiding precision medicine approaches and patient-tailored therapeutic strategies.

This study has several strengths. The integration of clinical phenotyping with real-time functional assays provides a dynamic understanding of disease mechanisms beyond static biomarker measurements. The use of CDS enabled direct characterization of ligand–receptor interactions, revealing a dynamic antagonism between irisin and TSP-1. In addition, the study is based on a well-characterized cohort diagnosed according to the Canadian Consensus Criteria, ensuring clinical relevance to hallmark features such as PEM. The inclusion of a standardized mechanical stress challenge further enabled the identification of impaired irisin responsiveness under physiologically relevant conditions. Importantly, multivariable regression analyses and appropriate statistical corrections for multiple testing strengthened the robustness of the findings by demonstrating that the association between irisin and fatigue severity is independent of key confounding variables. Furthermore, sex-stratified analyses revealed biologically relevant differences in the relationship between irisin signaling and symptom severity, highlighting potential sex-specific mechanisms in ME. However, several limitations should be acknowledged. In particular, the relatively small number of male participants limits the ability to formally assess sex-specific interaction effects. Future studies involving larger and more sex-balanced cohorts will, therefore, be important to better define the relationship between sex, irisin signaling, symptom severity, and metabolic dysfunction in ME. The cross-sectional design limits causal inference regarding the relationship between circulating irisin levels and disease progression. While Jurkat cells provide a controlled model for studying receptor-level interactions, they may not fully recapitulate the tissue-specific complexity of skeletal muscle, vascular endothelium, or central nervous system signaling in ME. Additionally, although the entire cohort was assessed at baseline, only a subset completed the mechanical stress challenge, which may limit the generalizability of the findings on exertional responses. Furthermore, the precise structural mechanisms underlying TSP-1 interference with the irisin–αvβ5–HSP90α complex remain to be elucidated. In addition, the present study did not evaluate upstream transcriptional regulators of FNDC5/irisin expression, including PGC1α, PPARα, or PPARγ, nor did it assess potential epigenetic regulation of the FNDC5 locus. Future studies integrating transcriptional, proteomic, and epigenetic approaches, including ChIP-based analyses, will be important to determine whether altered upstream regulatory mechanisms contribute to impaired irisin biology in ME. Such studies will also help clarify whether the observed dysregulation reflects altered gene expression, defective metabolic adaptation, or downstream signaling resistance mechanisms. Future studies incorporating longitudinal designs, primary human cell systems, and multi-omics approaches will be essential to validate these findings and further define the therapeutic potential of targeting the irisin–TSP-1 axis.

## 4. Materials and Methods

### 4.1. Study Population and Clinical Characterization

This study was designed as a cross-sectional analysis to investigate the molecular interplay between irisin and TSP-1 in the pathophysiology of ME. A total of 136 participants were recruited, comprising 92 ME patients and 44 sedentary HCs. All ME patients were diagnosed according to the CCC, which emphasizes the presence of hallmark symptoms such as PEM, persistent fatigue, cognitive dysfunction, and sleep disturbances [34]. HCs were sedentary individuals with no history of chronic fatigue or related disorders, matched to the ME cohort for age, sex, and BMI to ensure comparability. Written informed consent was obtained from all participants, and all procedures were approved by the Institutional Review Board of CHU Sainte-Justine (Comité D’Éthique du CHU Sainte-Justine, Project #4047). All experiments were performed in accordance with relevant guidelines and human ethics regulations.

Inclusion criteria for ME patients included a confirmed diagnosis according to CCC and an age range of 18 to 75 years. HCs were required to maintain a sedentary lifestyle. They were excluded if they reported any chronic conditions with symptoms resembling ME or a family history of ME or related conditions like fibromyalgia. Participants in both groups were excluded if they were pregnant or breastfeeding at the time of the study. Detailed clinical histories, including medication use, comorbidities, and prior medical conditions, were systematically collected by trained nursing staff at the time of the clinical visit using standardized questionnaires. Reported comorbidities within the ME cohort included cardiovascular/autonomic, endocrine/metabolic, gastrointestinal/hepatic, respiratory/allergic, neurological/neuropsychiatric, and autoimmune/inflammatory disorders, including fibromyalgia. Medication exposure included antihypertensive agents, corticosteroids, hormone-related therapies, antidepressants, gastrointestinal medications, analgesics, gabapentinoids, vitamin supplementation, and sleep-related medications. Because several of these medication classes have previously been associated with modulation of inflammatory, vascular, metabolic, or fibrotic pathways related to TSP-1 biology, subgroup analyses were performed to evaluate their potential influence on circulating TSP-1 levels. Furthermore, the study utilized a repeated-measures within-subject design based on a standardized physiological provocation maneuver with paired baseline and post-stress sampling. This approach minimizes the influence of stable inter-individual confounding variables, including illness duration, baseline physiological variability, and polypharmacy.

Clinical assessments were conducted using validated questionnaires to quantify symptom burden and functional status. SF-36 was used to evaluate physical and mental health components, while DSQ captured specific symptoms, including PEM, sleep disturbances, autonomic dysfunction, and cognitive impairments. Weekly physical activity levels were estimated by asking participants to report the number of hours spent in physical activity during the preceding week, using DSQ question 89.

Disease severity within the ME cohort was rigorously evaluated and stratified using the MFI-20, which measures dimensions of fatigue, including general fatigue, physical fatigue, reduced activity, reduced motivation, and mental fatigue [2,35]. For the purpose of level stratification, patients were categorized based on their total MFI-20 scores: 51–75 indicated mild fatigue, and 76–100 indicated moderate-to-severe fatigue. Additionally, the severity of PEM was specifically quantified using the validated DSQ-PEM subscale [1]. This multi-tiered assessment enabled correlation of circulating markers with both the subjective intensity of fatigue and the hallmark physiological response to exertion characteristic of the disease.

### 4.2. Post-Exertional Stress Challenge and Mechanical Stimulation Protocol

To evaluate the dynamic physiological response to exertion, a subset of the recruited cohort underwent a standardized 90 min mechanical stimulation protocol designed to mimic the physiological stress of physical activity [1]. While baseline venous blood samples were collected from the entire study population (92 ME patients and 44 HCs), the longitudinal post-exertional stress challenge and subsequent blood collection at T90 were completed by 52 ME patients and 31 HCs. This standardized protocol involved applying intermittent pneumatic compression to the upper arm with a device calibrated to deliver cyclic pressure variations (frequency: 0.006 Hz; pressure range: 0–4 psi). This specific frequency and pressure were selected to stimulate hemodynamic and vascular responses that partially recapitulate the physiological effects of physical exertion. Importantly, this model was designed to reproduce key circulatory and mechanotransductive components of exercise while minimizing confounding factors such as systemic metabolic exhaustion, excessive cardiovascular strain, and variability in physical performance. This approach is particularly relevant in ME populations, where conventional exercise testing may exacerbate symptoms and introduce significant inter-individual variability. Moreover, the use of a low-burden, non-exertional stimulation paradigm facilitates participation of patients with higher symptom severity who may otherwise be underrepresented in exercise-based studies.

### 4.3. Blood Collection and Plasma Preparation

Blood samples were collected in EDTA-coated tubes at both baseline (T0) and immediately after the application of the stress test (T90). This longitudinal design enabled precise calculation of the delta irisin (∆T90–T0), representing the absolute change in circulating irisin concentration and serving as a primary indicator of the systemic capacity to modulate myokine signaling under stress. Immediately following collection, samples were centrifuged at 216× *g* for 10 min at room temperature to separate the plasma fraction. Plasma aliquots were harvested and stored at −80 °C to preserve molecular stability until the time of determination. Following the initial plasma separation step, samples underwent an additional centrifugation at 10,000× *g* for 10 min at 4 °C prior to TSP-1 ELISA quantification, in accordance with the manufacturer’s recommendations, to minimize residual platelet contamination and cellular debris.

### 4.4. Quantification of Circulating Irisin and TSP-1

Circulating levels of irisin and TSP-1 were quantified in plasma samples using commercially available enzyme-linked immunosorbent assay (ELISA) kits according to the manufacturers’ instructions. Plasma irisin was measured using the Human irisin ELISA kit (MyBioSource, San Diego, CA, USA), while plasma TSP-1 levels were determined using the Quantikine sandwich ELISA immunoassay (R&D Systems, Minneapolis, MN, USA). All assays were performed in duplicate to ensure analytical reproducibility. Plasma samples were diluted to the desired concentration (typically 1:10 for irisin and 1:100 for TSP-1) to ensure that measured values fell within the linear range of the standard curve. Optical density (OD) was measured at 450 nm using a DTX880 Multimode Detector (Beckman Coulter, Brea, CA, USA). Absolute concentrations of irisin (µg/mL) and TSP-1 (ng/mL) were determined by interpolating OD values against standard curves generated using a four-parameter logistic (4-PL) regression model. All plasma samples were processed under standardized conditions and stored at −80 °C until analysis. Samples were aliquoted immediately after processing to minimize repeated freeze–thaw cycles, and all biomarker measurements were performed using first-thaw aliquots whenever possible. Duplicate measurements were performed for all samples, and only values within the linear range of the standard curves were included in the analysis. According to the manufacturers’ specifications, the intra-assay and inter-assay coefficients of variation were <10% and <12%, respectively, for both ELISAs.

### 4.5. Functional Signaling Analysis via Cellular Dielectric Spectroscopy (CDS)

Functional molecular interactions and receptor dynamics were assessed using a label-free, real-time microfluidic bioimpedance platform (CellKey™, MDS Sciex, San Francisco, CA, USA), as previously described [33]. Jurkat cells (human immortalized T lymphocytes) were selected as the cellular model due to their well-characterized signaling responses and their suitability for real-time bioimpedance-based analysis of receptor-mediated signaling dynamics. While this model enables controlled interrogation of ligand–receptor interactions, it may not fully recapitulate the tissue-specific complexity of skeletal muscle, vascular, or neuronal systems relevant to ME. Cells were cultured in RPMI-1640 medium (Wisent, Saint-Bruno, QC, Canada) supplemented with 10% fetal bovine serum (FBS), 1% penicillin–streptomycin, and 1% L-glutamine (Thermo Fisher Scientific, Waltham, MA, USA). Cell density and viability were assessed prior to each experiment using a CytoSmart cell counter (Corning Inc., Corning, NY, USA), with a minimum viability threshold of 80%.

CellKey™ 96-well microplates were preconditioned with 5 µL of unsupplemented RPMI and centrifuged at 216× *g* for 3 min to ensure uniform liquid distribution. Cells were seeded at a density of 2.5 × 10^4^ cells per well and incubated overnight at 37 °C in a humidified atmosphere containing 5% CO_2_. For dose–response analyses, cells were stimulated with recombinant human irisin (MyBioSource) or recombinant TSP-1 (R&D Systems) across a concentration range of 10^−9^ to 10^−5^ M. To assess reciprocal antagonism and signaling crosstalk, co-treatment experiments were performed in which cells were pretreated with one ligand (e.g., 10^−6^ M TSP-1) for 30 min prior to stimulation with the second ligand (e.g., 10^−6^ M irisin). To evaluate pathway specificity, cells were pre-incubated for 60 min with neutralizing antibodies targeting either HSP90α or αvβ5 integrin (Thermo Fisher Scientific; R&D Systems) before ligand stimulation. Isotype-matched IgG antibodies were used as negative controls to account for non-specific antibody effects. Vehicle-treated cells (culture medium alone) were included as baseline controls, and all responses were normalized to vehicle conditions. Receptor specificity was further supported by the selective attenuation of irisin-induced responses following αvβ5 or HSP90α blockade, while TSP-1 responses remained comparatively less affected under the same conditions. Real-time changes in bioimpedance, reflecting integrated cellular responses, were recorded for 15 min post-stimulation. Data were analyzed in kinetic mode, normalized to untreated controls, and expressed as a percentage of the maximal response to enable standardized comparisons across conditions. All experiments were conducted in triplicate to ensure reproducibility and analytical robustness.

### 4.6. Statistical Analysis

All statistical analyses were performed using GraphPad Prism (v9.0, GraphPad Software, La Jolla, CA, USA). Data normality was assessed using the Shapiro–Wilk test. Normality testing was performed separately for subgroup analyses prior to the application of parametric statistical tests, and non-parametric alternatives were considered when distribution assumptions were not met. Homogeneity of variance between groups was also evaluated prior to applying parametric independent *t*-tests and ANOVA analyses. Continuous variables are presented as mean ± standard error of the mean (SEM) for normally distributed data, or as median with interquartile range (IQR) for non-normally distributed data. Between-group comparisons of circulating molecular markers (ME vs. healthy controls) were conducted using unpaired two-tailed Student’s *t*-tests. Within-group longitudinal changes in irisin levels during the mechanical stress challenge (baseline T0 vs. post-stress T90) were assessed using paired two-tailed *t*-tests. The exertional response was further quantified as Δirisin (T90–T0) and compared between groups using unpaired *t*-tests. The total MFI-20 score was used as the dependent variable, with baseline irisin, age, sex, BMI, and disease duration included as covariates to assess the independent association between circulating irisin levels and fatigue severity. Only participants with complete datasets for all variables included in the regression model were retained for the final multivariable analysis. Effect sizes for group comparisons were calculated using Cohen’s d. For functional CDS experiments, concentration–response relationships were modeled using nonlinear regression (four-parameter logistic model). Differences between experimental conditions (e.g., vehicle vs. antibody blockade) and interactions between irisin and TSP-1 were evaluated using analysis of variance (ANOVA), followed by Tukey’s post hoc multiple comparisons tests. Associations between circulating irisin levels, symptom severity (DSQ-PEM), and physical activity were assessed using Pearson or Spearman correlation coefficients, as appropriate based on data distribution. To account for multiple testing in correlation analyses, *p*-values were adjusted using the Benjamini–Hochberg false discovery rate (FDR) correction. For analyses involving multiple comparisons, including correlation analyses and subgroup evaluations, *p*-values were corrected using the Benjamini–Hochberg procedure to control the false discovery rate. Both raw and FDR-adjusted *p*-values are reported where applicable. Sex-stratified analyses were conducted as exploratory (post hoc) analyses. Outlier analysis was formally conducted using the ROUT method (Q = 1%); no outliers were identified, and all data points were retained in the final analyses. All statistical tests were two-tailed, and significance was set a priori at *p* < 0.05. Exact *p*-values are reported where possible. Statistical significance in figures is denoted as follows: *p* < 0.05, *p* < 0.01, *p* < 0.001, and *p* < 0.0001.

## 5. Conclusions

In conclusion, this study identifies a significant dysregulation of the irisin–TSP-1 signaling axis in patients with ME. We show that ME is characterized by reduced baseline circulating irisin levels and a markedly blunted response to exertional stress, highlighting an impaired capacity to regulate a key metabolic myokine. This dysregulation provides a potential biological basis for the disrupted energy homeostasis and bioenergetic inflexibility observed in this population. Our findings further reveal a clinical paradox: although irisin levels are reduced at the group level, baseline irisin remains an independent predictor of fatigue severity. This relationship is accompanied by a parallel, severity-dependent increase in the antagonist TSP-1, suggesting a shift toward a state of signaling resistance. Mechanistically, functional analyses using CDS demonstrate that TSP-1 is associated with inhibition of irisin signaling within a signaling context sensitive to αvβ5 blockade and HSP90α inhibition. This antagonistic interaction supports a model of an impaired metabolic adaptability in which compensatory increases in irisin in more severely affected patients fail to restore metabolic function due to concurrent upregulation of TSP-1. Collectively, these findings highlight the irisin–TSP-1 axis as a critical regulator of metabolic signaling in ME and suggest that therapeutic strategies aimed at restoring this balance may represent a potential avenue that warrants further validation. In addition, irisin–TSP-1 profiling may offer a clinically relevant approach for stratifying metabolic endophenotypes in ME and guiding precision medicine strategies.

## Figures and Tables

**Table 1:** Demographic and clinical characteristics of study participants.

Characteristic	ME (*n* = 92)	HCs (*n* = 44)	*p*-Value
Women/Men	71/21	25/19	NS
Age (years)	49 ± 1.2	50 ± 1.6	NS
BMI (kg/m^2^)	26 ± 0.6	25 ± 0.7	NS
Illness Duration (years)	15 ± 1.3	N/A	-
**SF-36 SCORE**			
Physical Health	34 ± 1.5	89 ± 1.6	<0.0001
Mental Health	44 ± 2.0	88 ± 1.8	<0.0001
**MFI-20 SCORE**			
General Fatigue	18 ± 0.3	7 ± 0.4	<0.0001
Physical Fatigue	17 ± 0.3	6 ± 0.4	<0.0001
Reduced Activity	16 ± 0.4	6 ± 0.4	<0.0001
Reduced Motivation	11 ± 0.4	6 ± 0.3	<0.0001
Mental Fatigue	15 ± 0.4	7 ± 0.5	<0.0001
**DSQ SCORE**			
Autonomic Neuroendocrine	40 ± 1.7	7 ± 1.0	<0.0001
Cognitive Dysfunction	59 ± 2.0	11 ± 1.8	<0.0001
Post-Exertional Malaise (PEM)	66 ± 2.1	8 ± 1.2	<0.0001
Sleep Score	51 ± 1.7	14 ± 1.5	<0.0001

Demographic and clinical characteristics of patients with Myalgic Encephalomyelitis (ME) compared to Healthy Controls (HCs). Data are presented as mean ± standard error for continuous variables (age, body mass index (BMI), illness duration) and as counts for categorical variables. ‘*n*’ represents the number of participants in each group. BMI: Body Mass Index; N/A: not applicable.

**Table 2:** Multiple Linear Regression Analysis Identifying Independent Predictors of Fatigue Severity (MFI-20) in ME Patients. Statistical significance is indicated by * *p* < 0.05, ** *p* < 0.0001.

Variable	Coefficient (β)	Standard Error	95% CI	t-Ratio	*p*-Value	VIF
Intercept	62.21	9.52	43.26 to 81.16	6.532	<0.0001 **	
Irisin (μg/mL) T0	0.67	0.284	0.106 to 1.235	2.361	0.021 *	1.15
BMI	0.206	0.219	−0.230 to 0.643	0.941	0.35	1.02
Sex (1 = F, 2 = M)	−2.2	2.996	−8.161 to 3.761	0.734	0.465	1.12
Age	0.026	0.121	−0.215 to 0.268	0.216	0.829	1.38
Disease Duration	0.017	0.116	−0.213 to 0.247	0.144	0.886	1.35

## Data Availability

The data supporting the findings of this study have been provided as part of the journal submission files and are available from the corresponding author upon reasonable request.

## Abbreviations

The following abbreviations are used in this manuscript:

BMI	Body Mass Index
CCC	Canadian Consensus Criteria
CDS	Cellular Dielectric Spectroscopy
CI	Confidence Interval
DSQ	DePaul Symptom Questionnaire
FNDC5	Fibronectin Type III Domain-Containing Protein 5
HC	Healthy Control(s)
HSP90	Heat Shock Protein 90
ME	Myalgic Encephalomyelitis
MFI-20	Multidimensional Fatigue Inventory
NO	Nitric Oxide
PEM	Post-Exertional Malaise
PGC1-α	Peroxisome Proliferator-Activated Receptor Gamma Coactivator 1-Alpha
SF-36	Short Form-36 Health Survey
T0	Baseline Time Point
T90	Post-Stress Time Point (90 min)
TSP-1	Thrombospondin-1
VIF	Variance Inflation Factor
αvβ5	Alpha V Beta 5 Integrin
Δ (Delta)	Change between Time Points
